# Total CAD/CAM Supported Method for Manufacturing Removable Complete Dentures

**DOI:** 10.1155/2016/1259581

**Published:** 2016-11-16

**Authors:** Arthur Furtado de Mendonça, Mario Furtado de Mendonça, George Shelby White, Georges Sara, Darren Littlefair

**Affiliations:** ^1^Department of Prosthodontics, Fluminense Federal University, Niterói, RJ, Brazil; ^2^Department of Prosthodontics, Columbia University College of Dental Medicine, New York, NY, USA; ^3^University of Manchester (Formerly UMIST), Manchester, UK

## Abstract

The incorporation of computer-aided design/computer-aided manufacturing (CAD/CAM) technology into complete denture fabrication brings about several advantages to the fabrication process, providing better predictability of the desired outcomes and high accuracy of denture fit, mainly because the milling of prepolymerized acrylic resin eliminates the shrinkage of the acrylic base. Also, there is a decrease in the porosity when compared to a conventionally processed denture, and consequently there is a decrease in the retention of* Candida albicans* on the denture base. The presented workflow for complete denture fabrication presents a totally wax-free manufacturing process, combining rapid prototyping (RP) and rapid milling. With the presented technique, the maxillomandibular relation (MMR) and the ideal setup of the tooth arrangement are developed by using occlusion rims and trial setup made with RP. For the definitive final denture, the denture base and the basal surfaces of the conventional denture teeth were milled according to the individual clinical situation. Posteriorly, the teeth were adapted and bonded into the milled sockets of the milled base.

## 1. Introduction

Since the introduction of computer-aided design/computer-aided manufacturing (CAD/CAM) techniques, considerable changes have taken place in dentistry; fixed dental prostheses have been extensively reported, while clinical reports of total CAD/CAM workflow for complete removable dentures are scarce. Some systems are available for the fabrication of removable dentures, including milling and rapid prototyping, but still some limitations and disadvantages can be found, such as manufacturing challenges caused by making impression, establishing occlusal vertical dimension (OVD), maxillomandibular relation (MMR) transfer, inability to define the mandibular occlusal plane, expensive materials, and increased laboratory costs compared with those for conventional methods [1]. Also, some systems do not provide a trial denture, which is considered an important step in validating comfort, function aesthetics, and patient acceptance before the final fabrication is issued to the patient.

Current innovations and developments in dental technology address these limitations and allow the fabrication of removable dentures by using CAD/CAM from start to finish, achieving a less traumatic experience for the patients for better fitting dentures. The first step for manufacturing the digital dentures is to prepare the casts using conventional impression techniques or intraoral digital impression; therefore, casts are scanned and the MMR is registered using occlusion rims manufactured using RP. Stereolithography (STL) files with the appropriate OVD and MMR are generated with the scans of the occlusion rims after the interocclusal records, tooth selection, and arrangement. Subsequently, the trial denture is 3D printed using RP and the clinical parameters are evaluated, so the final denture can be milled.

The presented report of a clinical study provides a detailed description of a total CAD/CAM supported method for manufacturing removable complete dentures. The workflow is managed using the Prosthetic Design Centre software (PDC, Stoneglass Industries).

## 2. Case Presentation

**Figure 1:**
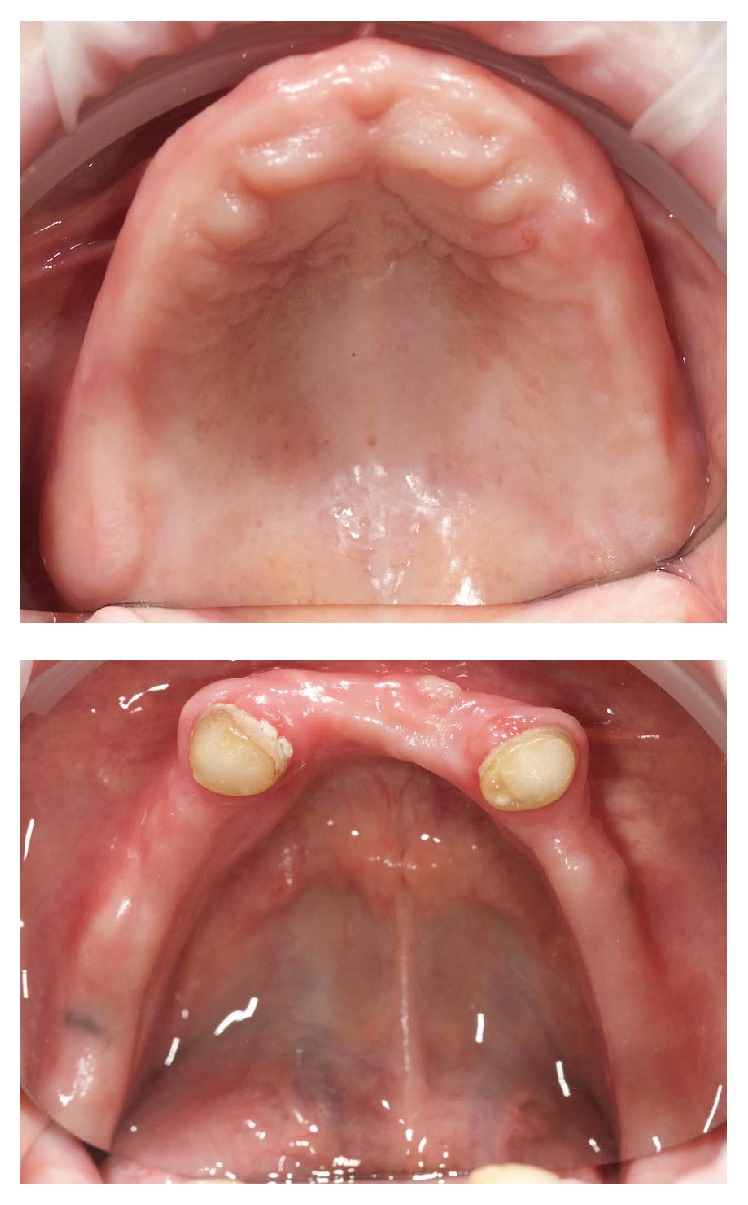
Clinical condition.

**Figure 2:**
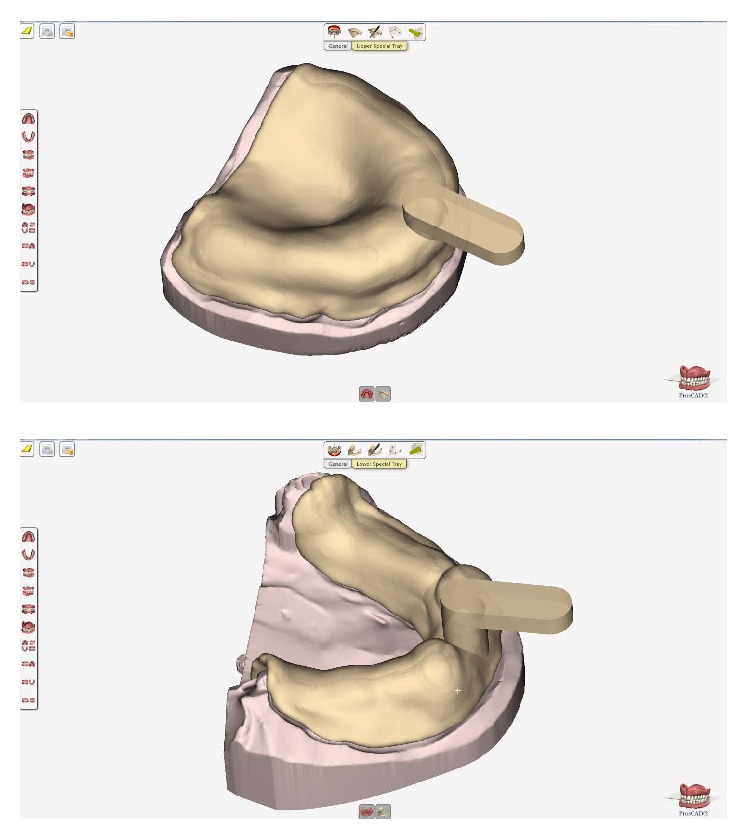
Design of special trays.

**Figure 3:**
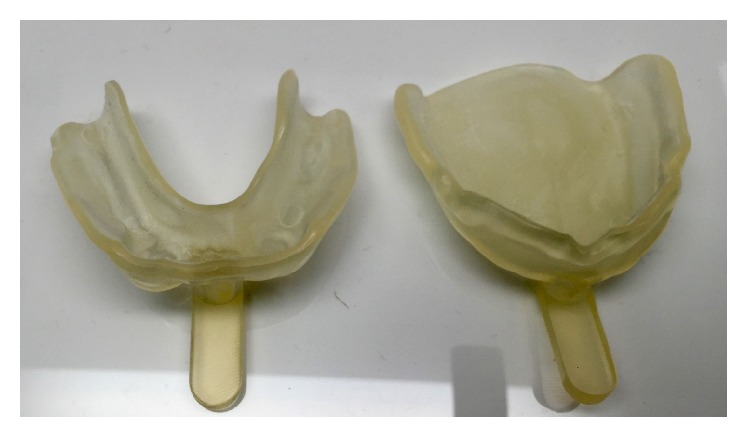
Special trays manufactured by rapid prototyping.

**Figure 4:**
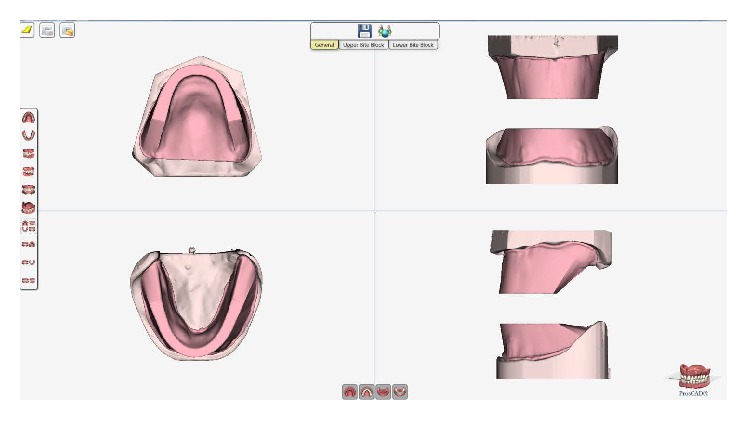
Digital design of the occlusal rims.

A 63-year-old woman presented without any significant medical problems to replace her current removable denture. On the mandibular arch, the prepared teeth 33 and 34 were presented (Figure 1). For the primary casts, conventional impressions using alginate (Alginoplast, regular set; Heraeus Kulzer GmbH) were made for subsequent scanning and digital designed special trays (first clinical appointment). The correct path of insertion and the extension and width of the trays were designed and managed within the PDC software. The trays were manufactured with rapid prototyping process (ProJet MP 3500, 3D Systems Inc., USA) (Figures 2 and 3). Once the definitive impressions (Impregum Penta; 3M ESPE) of the maxilla and mandible were completed with the 3D printed special trays (second clinical appointment), the secondary casts were fabricated, which then are scanned for the digital design of the occlusal rims (Figure 4).

**Figure 5:**
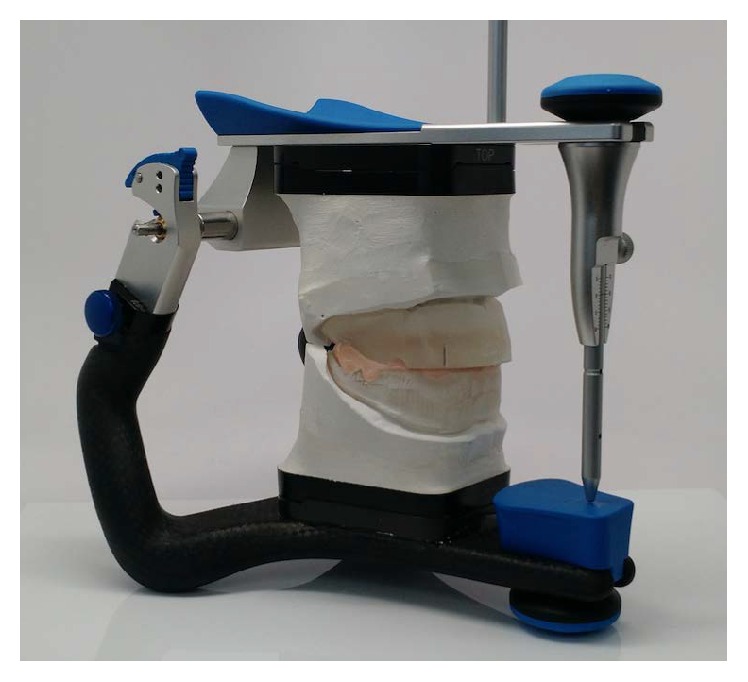
Record of the maxillomandibular relation.

The third clinical appointment is dedicated to adjusting the occlusion rims and recording the clinical data following the basic protocol for removable dentures, like the vertical dimension of occlusion, smile line, the position of the canines, and the midline. When the occlusion rims were articulated properly, the MMR was registered with a bite registration material (Blu-Mousse, Parkell Inc., USA) (Figure 5) and scanned for the digital design of teeth and gingival tissues. The scan of the MMR was made in two steps: first, the casts were mounted on an articulator (Artex, Amann Girrbach, USA) and then the casts were scanned separately with an optical scanner (IScan d104i, Imetric 3D Scanning Systems, Switzerland), and with a precalibrated device the MMR was transferred to a STL file.

**Figure 6:**
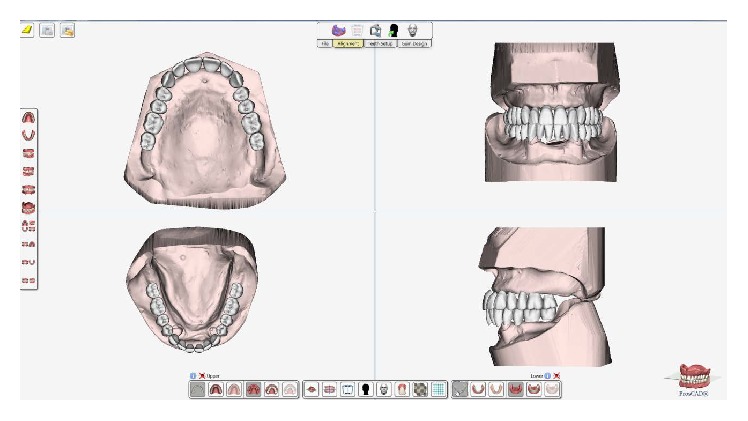
Digital design of the teeth.

**Figure 7:**
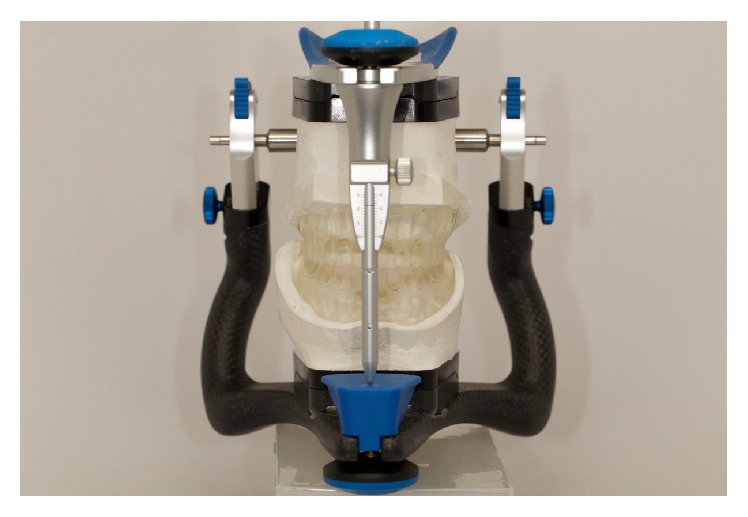
First prototype.

**Figure 8:**
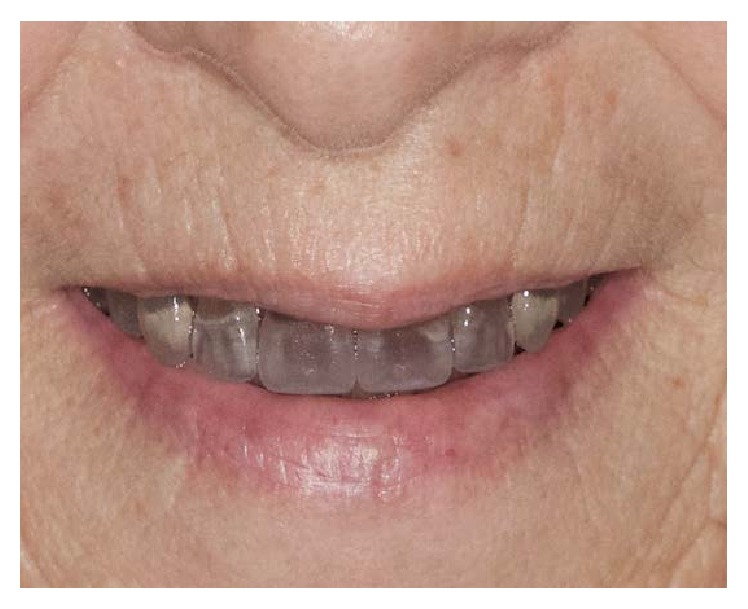
Clinical evaluation of the first prototype.

**Figure 9:**
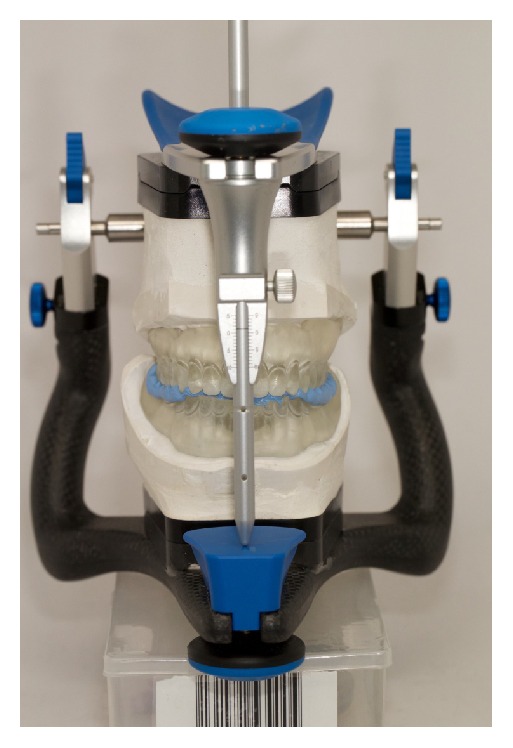
Bite record after the primary changes on the occlusion plane.

**Figure 10:**
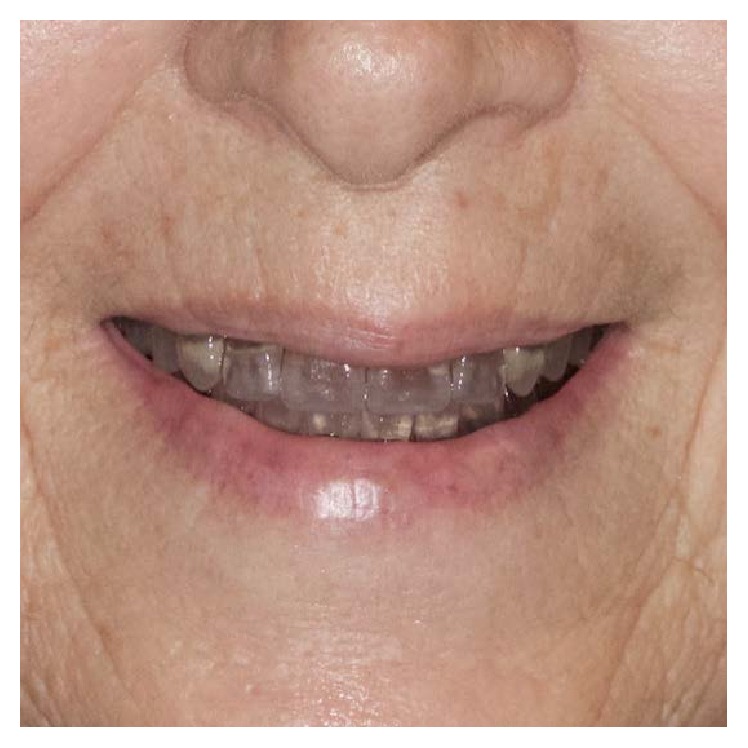
Second prototype.

Following the PDC workflow, tooth selection and arrangement can be accomplished by using the data on the adjusted occlusion rims for appropriate lip support and optimal aesthetics (Figures 6 and 7). The first prototype is then 3D printed (ProJet MP 3500, 3D Systems Inc., USA) for the patient's trial setup. After making proper adjustments to the RP clinical setup, occlusal landmarks can be validated (fourth clinical appointment). Adjustments were necessary on the plane of occlusion and the MMR. Final adjustments can be made if necessary (Figures 8 and 9). After the clinician has verified the occlusal data and patient acceptance has been established (Figure 10), the final prosthesis can be sent for milling and are ready to be issued.

**Figure 11:**
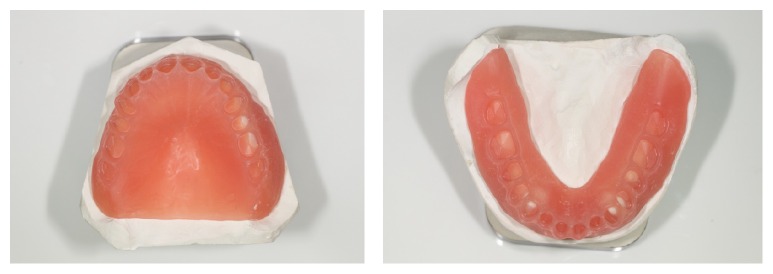
Denture bases.

**Figure 12:**
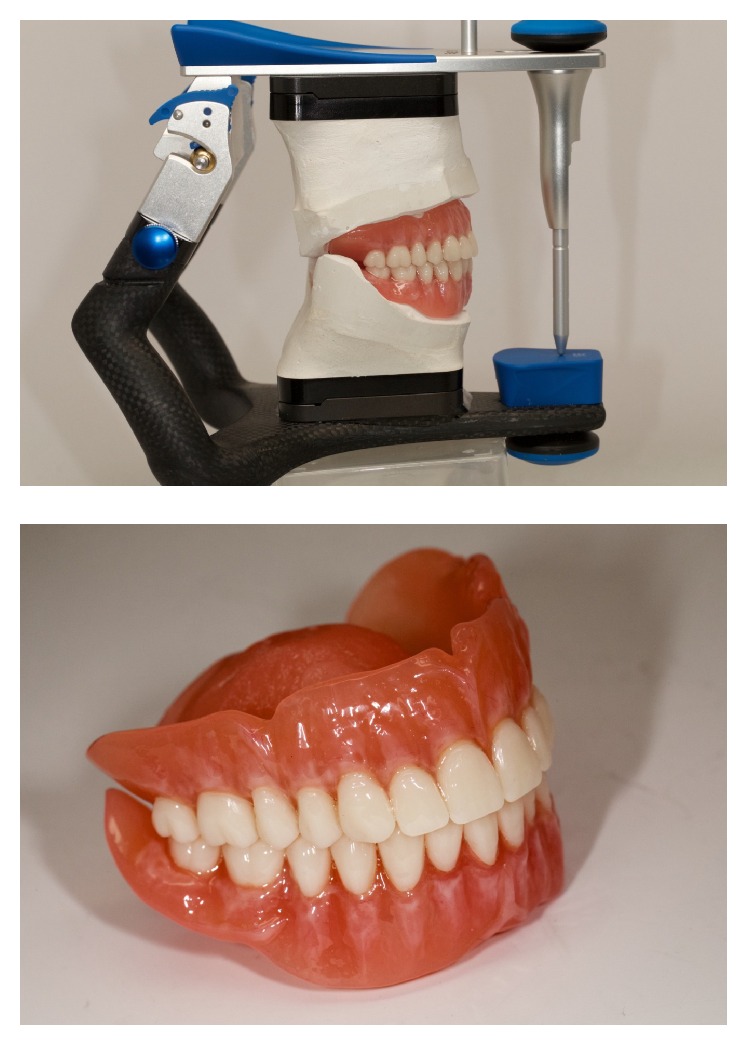
Final denture.

**Figure 13:**
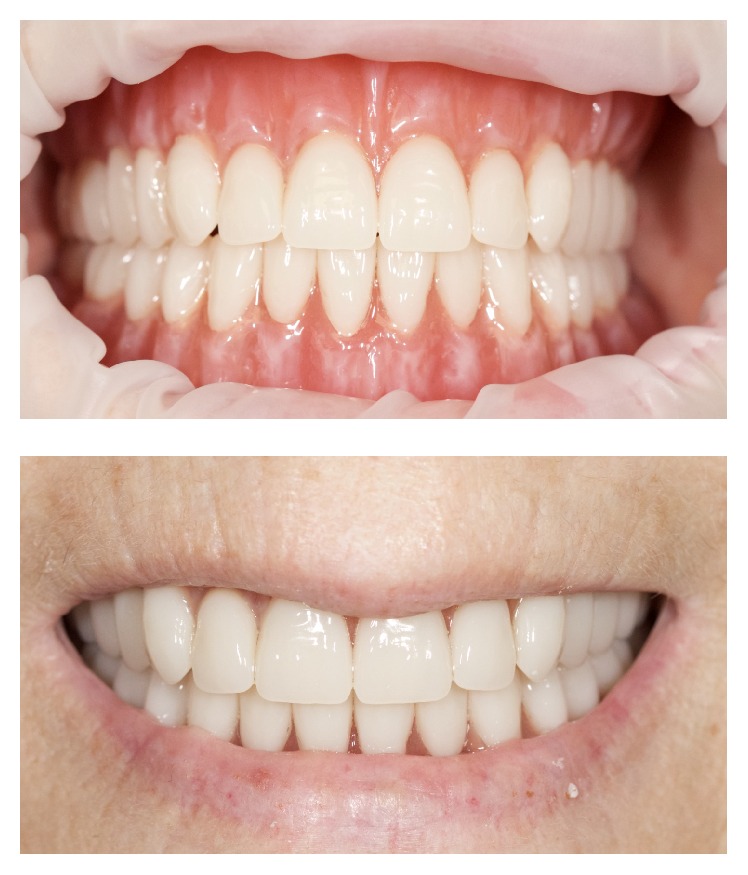
Clinical photos of the final denture.

The base of the denture is ready to be manufactured by milling a poly(methyl methacrylate) (PMMA) block with the information provided by the CAD software. Milling will provide individual sockets for each tooth (Figure 11). The conventional denture teeth (Heraeus Kulzer GmbH, Germany) were modified according to the individual clinical situation. For that purpose, they were mounted in a special device and had their basal surfaces milled according to prior computation. The teeth were bonded in the milled sockets of the denture base by mechanic retention done in the milling of the denture tooth (each tooth was milled according to its respective socket on the denture base with additional physical retention), and also chemical bonding was obtained by acrylic and monomer activation (Heraeus Kulzer GmbH, Germany). Denture base was stained with Heraeus Kulzer Pala cre-active Stains. The complete denture was polished and delivered (Figures 12 and 13). No adjustments were made in the delivery of the denture, nor was any follow-up necessary, demonstrating the accuracy of the technique (the final denture was a precise replica of the trial, which was approved by the clinician and the patient).

## 3. Discussion

In the past few years, significant advancements have taken place in the fabrication of complete dentures with the introduction of CAD/CAM technologies. The digital workflow was observed to provide better control of the desired outcomes and also improved the communication between all three parties consisting of the dentist, the patient, and laboratories. It is important to highlight the basic principles for the rehabilitation of the edentulous patient with removable dentures using CAD/CAM technologies and to continue to follow the same principles used to manufacture the analog dentures. With digital dentures, some of the steps that led to failure can be eliminated, like the polymerization shrinkage of the dental base, providing better predictability of the results, high accuracy in denture fit, and easier duplication of dentures [2–5].

The use of CAD/CAM to support the manufacture of removable dentures has been previously reported with different concepts. Some techniques use wax to make the occlusion rims and mill the denture bases with a wax blank for the trial evaluation in the patient mouth. A conventional processing technique is necessary [6]. Another reported system uses a wax occlusion rim for record bases and an anatomic measurement device to complete the OVD record, including centric relation [2]. This technique claimed to decrease the number of clinical appointments but faces the disadvantage of lacking a trial denture, which is considered an important step validating denture goals by patients and dentists before final denture fabrication [1]. The PDC dentures are produced by machining a preformed cylinder of PMMA. This material presents a decrease in the porosity when compared to a conventionally processed denture and a decrease in the retention of* Candida albicans* on the denture base [4]. The conventional denture teeth have their basal surface milled individually and are then bonded chemically to the corresponding sockets.

The PDC workflow presents unique characteristics, because it is a totally wax-free system, which utilizes rapid prototyping to manufacture the occlusion rims, the record of the clinical parameters, and the trial setup for the evaluation of the final design. In this particular case, the first trial, after verifying the occlusion and the eccentric movements, it was decided to make some changes on the tooth position to improve the function and aesthetics. The trial setup was adjusted and a new bite record was made for posterior scanning. The second trial was considered the desirable setup by all the parties involved. The final denture was issued, and at the final appointment no modification was needed on the dentures, demonstrating that successful criteria had been met.

## 4. Conclusions

A totally wax-free technique for the fabrication of a CAD/CAM denture was described. By using RP, a trial made it possible for all parties to evaluate criteria for successes before the final denture is issued providing control of the outcomes. This clinical report demonstrates that digital denture can be effective and accurate, eliminating or replacing steps that can lead to complications. Since this is a novel approach, further research should provide the limitations of the technique in terms of color stability, stain techniques, and teeth/denture base bond strength. A controlled randomized clinical trial is needed to determine whether CAD/CAM digital dentures are an improvement over conventional methods for removable dentures.
